# Modular combinatorial binding among human *trans*-acting factors reveals direct and indirect factor binding

**DOI:** 10.1186/s12864-016-3434-3

**Published:** 2017-01-06

**Authors:** Yuchun Guo, David K. Gifford

**Affiliations:** MIT, Computer Science and Artificial Intelligence Laboratory, Cambridge, MA 02139 USA

**Keywords:** Computational genomics, Transcription factor, Combinatorial binding, Direct and indirect binding, Topic model

## Abstract

**Background:**

The combinatorial binding of *trans*-acting factors (TFs) to the DNA is critical to the spatial and temporal specificity of gene regulation. For certain regulatory regions, more than one regulatory module (set of TFs that bind together) are combined to achieve context-specific gene regulation. However, previous approaches are limited to either pairwise TF co-association analysis or assuming that only one module is used in each regulatory region.

**Results:**

We present a new computational approach that models the modular organization of TF combinatorial binding. Our method learns compact and coherent regulatory modules from in vivo binding data using a topic model. We found that the binding of 115 TFs in K562 cells can be organized into 49 interpretable modules. Furthermore, we found that tens of thousands of regulatory regions use multiple modules, a structure that cannot be observed with previous hard clustering based methods. The modules discovered recapitulate many published protein-protein physical interactions, have consistent functional annotations of chromatin states, and uncover context specific co-binding such as gene proximal binding of NFY + FOS + SP and distal binding of NFY + FOS + USF. For certain TFs, the co-binding partners of direct binding (motif present) differs from those of indirect binding (motif absent); the distinct set of co-binding partners can predict whether the TF binds directly or indirectly with up to 95% accuracy. Joint analysis across two cell types reveals both cell-type-specific and shared regulatory modules.

**Conclusions:**

Our results provide comprehensive cell-type-specific combinatorial binding maps and suggest a modular organization of combinatorial binding.

**Electronic supplementary material:**

The online version of this article (doi:10.1186/s12864-016-3434-3) contains supplementary material, which is available to authorized users.

## Background

The combinatorial binding of *trans*-acting factors (TFs) is an important basis for the spatial and temporal specificity of gene regulation [1–4]. Combinations of TFs have been shown to regulate gene expression stripes in the Drosophila embryo [5], to generate cell-type-specific signaling responses [6, 7], and to program cell fates [8]. In this paper we call each distinct set of TFs that bind together to the same regulatory regions a *regulatory module*.

Understanding the interplay among regulatory modules is essential to dissect the complexity of gene regulation. Previous studies have found that TFs tend to bind in clusters, which are typically characterized by a large number of TF binding sites in a regulatory region [9–12]. These co-binding TFs may belong to different functional modules that can be combined in regulatory regions to achieve specific functions. For example, gene regulation is initiated by the interaction of enhancer-bound TFs, promoter-bound TFs, and TFs that bring the enhancers and promoters together in three dimensions, such as mediator, CTCF, and cohesin [13–15]. Therefore, CTCF/cohesin modules may co-occur with enhancer-related modules, promoter-related modules, or both. Such module co-occurrences suggest that combinatorial binding of TFs may be organized in a modular hierarchy: a regulatory region may use a combination of multiple regulatory modules, which are, in turn, combinations of multiple TFs.

However, previous methods for studying combinatorial binding do not consider the modular organization of TF co-binding. Early work in discovering TF co-binding was limited to lower organisms or to the computational prediction of motif sites [16–19]. The systematic discovery of regulatory modules in humans has recently become possible with large-scale efforts such as the ENCODE project to comprehensively profile the in vivo binding of tens to hundreds of TFs in multiple human cell types [20, 21]. Initial analyses of the ENCODE data were limited to either pairwise TF co-binding [20] or TF co-binding in the genomic regions bound by a particular TF [9] and thus did not allow comprehensive discovery of higher order combinatorial binding. Hard-clustering-based methods have been applied to genome-wide binding data. For example, self-organizing maps (SOMs) have been used to explore and visualize the colocalization patterns of TFs [21] and k-means clustering has been used to characterize the combinatorial regulation of erythroid enhancers [22]. These methods model TF binding at a given region with a single module and consequently require a large number of modules to fully represent the complexity of TF combinatorial binding. For example, Xie et al. applied SOM to a dataset from K562 cells and estimated the optimal number of neurons (or modules) for the resulting SOM to be 2,852 [21]. Non-negative matrix factorization (NMF), a soft clustering method, has also been applied to infer TF interactions [23]. However, this work did not explicitly explore the issue of multiple module usage and predicted only a small number of TF combinations [23]. It notably failed to capture the well-studied CTCF/cohesin interaction [14, 24]. Therefore, we have found that existing methods are not suitable for modeling the modular structure of multiple regulatory modules in the same regulatory regions.

The need to model the modular organization of regulatory modules motivated us to use a probabilistic topic model that can represent modular TF co-binding in regulatory regions. Topic models have been widely used to discover thematic structures in a large corpus of documents [25, 26]. A topic model decomposes documents into a set of all shared topics, where a topic is a set of words that co-occur in multiple documents. The factoring of a document into multiple topics permits the discovery of compact and coherent topics that can be combined to accurately represent a document. This factoring results in better performance in predicting held-out data than mixture models [26], which are hard-clustering methods that force a document to be described by a single topic. Topic modeling has been used to discover gene expression programs [27, 28] and microRNA regulatory modules [29], but it has not yet been applied to study TF combinatorial binding.

Regulatory Module Discovery (RMD) applies a topic model to systematically discover regulatory modules using a large compendium of in vivo TF binding data. We show that RMD discovers more compact and comprehensive modules than other methods. Applying RMD to data from human K562 cells, we discovered diverse sets of regulatory modules and found that tens of thousands of regulatory regions use multiple modules in a modular manner. We found that, for certain TFs, direct (motif present) and indirect (motif absent) binding of the TF associates with distinct sets of co-binding partners. Finally, our analysis discovered cell-type-specific modules and shared modules, and that a given regulatory region can utilize different modules in different cell types. Overall, our results provide comprehensive cell-type-specific global maps of regulatory modules and suggest a modular organization of TF combinatorial binding in regulatory regions.

## Results and discussion

### Regulatory module discovery (RMD) discovers more compact and comprehensive regulatory modules than other methods

RMD discovers regulatory modules given binding data for a large set of regulatory regions. RMD is based on Hierarchical Dirichlet Processes [30], a Bayesian non-parametric topic model that automatically determines the number of modules based on the complexity of the observed data. To use conventional document topic model terminology, regulatory regions are “documents,” TF binding sites are “words,” and regulatory modules are “topics.” As in the document model, a regulatory region may utilize one or more modules, and a TF may participate in multiple modules.

**Fig. 1:**
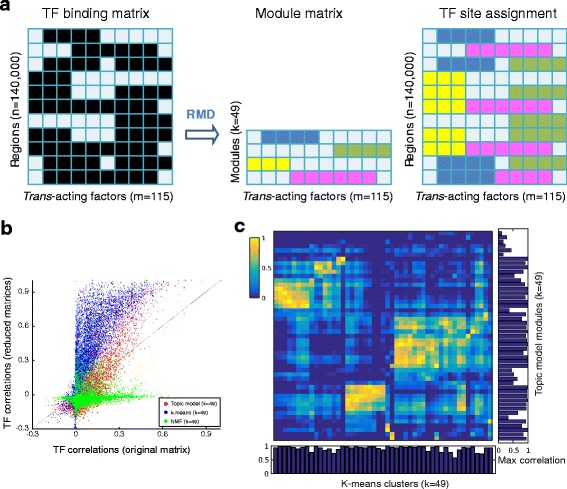
RMD discovers compact and comprehensive regulatory modules. **a** RMD discovers regulatory modules from regulatory regions across the genome. A region may use one or more modules. A factor may participate in one or more modules. RMD uses a topic model to summarize the binding data into modules and to assign binding sites to the modules. **b** The topic model re-capitulates the original binding data more accurately than k-means clustering and NMF. Each point in the scatter plot represents the Pearson correlation coefficient between a pair of TFs calculated using the original binding data (x-axis) or calculated using the reduced data matrix (k = 49) by topic model, k-means clustering, or NMF (y-axis). **c** A heatmap shows the Pearson correlations between topic model modules and k-means clusters (k = 49). The modules are ordered as in Fig. 2. Bottom bar chart shows the maximum correlation values for each k-means cluster. All k-means clusters are matched by at least one module, with a maximum correlation value larger than 0.5. Right bar chart shows the maximum correlation values for each module. Ten modules cannot be matched by any k-means clusters

The co-binding of TFs in regulatory regions across the genome can be represented without loss by a region-TF matrix. In the work below, a full region-TF matrix would be of size ~140,000 regions by 115 TFs and is difficult to directly interpret. Using topic modeling, a large region-TF matrix is summarized into a compact module-TF matrix (49 modules × 115 TFs) and a TF site assignment table (Fig. 1a). The module-TF matrix describes the set of regulatory modules discovered, with each module represented as a probability distribution over all the TFs. The assignment table assigns each TF binding site from each region to one of the modules. The assignment table can be further summarized into a region-module matrix (~140,000 regions × 49 modules) that describes which modules each regulatory region uses.

We first tested the ability of RMD, k-means clustering, and NMF to accurately capture TF-TF correlations that are present in the binding data. We used a compendium of ChIP-seq data from 115 TFs in human K562 cells [20], and then pooled and merged all the TF binding sites into ~140,000 non-overlapping co-binding regions, each of which was required to contain at least 3 TF binding sites. We applied the three methods to these ~140,000 co-binding regions and constrained them to discover the same number of modules (k = 49). Then for each method, we evaluated how well the pairwise TF correlation scores from the module-TF matrix correlate to those from the original region-TF matrix. We found that the topic model modules more accurately recapitulate the pairwise TF co-binding relationship in the original data (*r* = 0.81) than the modules learned from the other two methods (*r* = 0.74 for k-means clustering, *r* = 0.30 for NMF) (Fig. 1b). When the number of modules k is increased to 100, the performance of k-means clustering and NMF improves to *r* = 0.80 and *r* = 0.59, respectively (Additional file 1: Figure S1a). In addition, we observed that the k-means modules tend to be more similar to each other than those from RMD and those from the original binding data (Fig. 1b). Because hard-clustering-based methods do not factor complex binding regions into multiple modules, they generally generate more similar modules and need more modules to represent the structure in the data than a topic model. However, the increase in module count makes the interpretation of the modules harder. In the limit, increasing k to the total number of regions will exactly recapitulate the original binding data but will not permit common patterns of co-binding to be observed.

Given that RMD needs fewer modules to represent the binding data than k-means clustering, we then asked whether the RMD modules were sufficient to represent the clusters produced by k-means (k = 49 and k = 100). For this analysis, we considered an RMD module to match a k-means cluster and vice-versa if the Pearson correlation between the module/cluster vectors is greater than 0.5. We found that all the k-means clusters (k = 49) are matched by RMD modules, while 10 RMD modules are not matched by any k-means cluster for k = 49 (Fig. 1c). These unmatched RMD modules are used in small number of regions and often co-occur with other more widely used modules. For example, Module 4 (STAT1 + STAT2 + STAT5A) binds in 960 regulatory regions that are highly enriched with genes involved in immune response (FDR q-value = 2.2E-14) and Interferon alpha/beta signaling (FDR q-value = 2.2E-14), as shown by a GREAT analysis of annotation enrichment [31]. Two thirds of these regions also use other modules such as enhancer or promoter modules. Other modules that are not identified by k-means include co-binding of ZBTB33 [32] and promoter associated factors (Module 1), and of Pol3 + BDP1 + ATF3 (Module 17 and 41). Even when k is 100 for k-means, 6 RMD modules are not matched by any k-means cluster, while all the k-means clusters are matched by RMD modules (Additional file 1: Figure S1b). These comparison results are robust across different correlation cutoff values for matching the modules (Additional file 1: Figure S1c). The unmatched modules are missed by k-means clustering likely because they are over-shadowed by the other more widely used modules in the same regions.

In summary, our analysis shows that RMD is better at decomposing complex binding regions into a combination of specific modules and learning a set of more accurate, compact, and comprehensive modules than hard clustering and matrix factorization approaches.

### A global map of regulatory modules in human cells

**Fig. 2:**
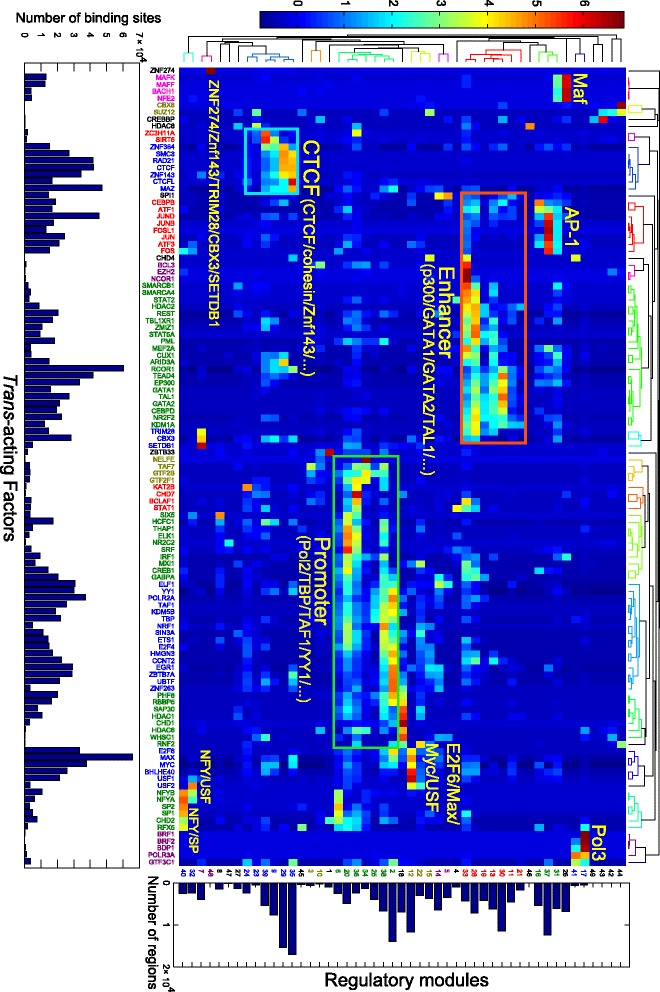
*Trans*-acting factors bind in a complex combinatorial and context-specific manner. RMD was applied to a compendium of ChIP-seq binding sites of 115 TFs (~140,000 regions) in human K562 cells and discovered 49 modules. Each cell in the heatmap represents the z-score of the TF binding site count (standardized along the columns) of a TF (*column*) in a module (*row*). The bottom bar plot shows the total number of binding sites of the TFs. The right bar plot shows the number of regions that use the modules. The top and left dendrograms were computed by applying hierarchical clustering on the regulatory module matrix with Pearson correlation distance and average linkage. The module and TF labels are colored according to the hierarchical clusters. Selected groups of similar modules are labelled based on the major TFs participating in each module

Applying RMD to the K562 dataset, we discovered a global combinatorial binding map consisting of 49 regulatory modules (Fig. 2 and Additional file 2: Tables S1-S2). We also applied RMD to data from GM12878 cells (86 TFs) and discovered 49 modules (Additional file 1: Figure S2 and Additional file 2: Tables S3-S4).

We found that the discovered modules are easy to interpret and reveal coherent functional groups of co-binding TFs. The modules discovered from K562 cells capture known sets of factors that interact with each other or function as a complex, such as the following:

- the master regulators GATA1, GATA2 and TAL1 [33]; and the enhancer-binding co-activator p300
- the transcriptional machinery Pol2, TBP, and TAF1; promoter-binding TFs such as E2F6 [34]; and transcription start site associated chromatin regulators such as PHF8 [35], etc.
- CTCF, cohesin subunits RAD21 and SMC3, and ZNF143 [13, 36]
- Pol3 transcriptional machinery [37]
- AP-1 factors such as JUN/JUNB/JUND/FOS/FOSL [38]
- MAF/BACH1/NFE2 [39]
- MYC/MAX/USF/E2F6 [40]
- SPI1 (also known as PU.1) and ELF1 [41]

To further evaluate whether the discovered modules were consistent with known TF interactions, we compared TF co-occurrences in these modules with published in vivo protein-protein interactions assayed by antibody immunoprecipitation and mass spectrometry (IP-MS) in K562 cells [21]. Because of the limited coverage of the IP-MS dataset, we were not able to evaluate the specificity of the TF combinations we discovered. Similar to the original study [21], we evaluated the sensitivity in recovering the IP-MS interactions using TF-TF associations discovered by RMD. Among the 115 TFs we studied, 33 physical protein-protein interactions were identified in the published IP-MS dataset, 22 of these TF interactions are captured as TF combinations in the K562 regulatory modules (*p* value < 0.05) (Additional file 3: Table S7). The fraction of overlap is similar to the original study, which discovered TF co-binding as 2,852 SOM neurons (i.e. modules) [21]. Thus, RMD is able to recapitulate previous findings with a much smaller number of interpretable modules.

**Fig. 3:**
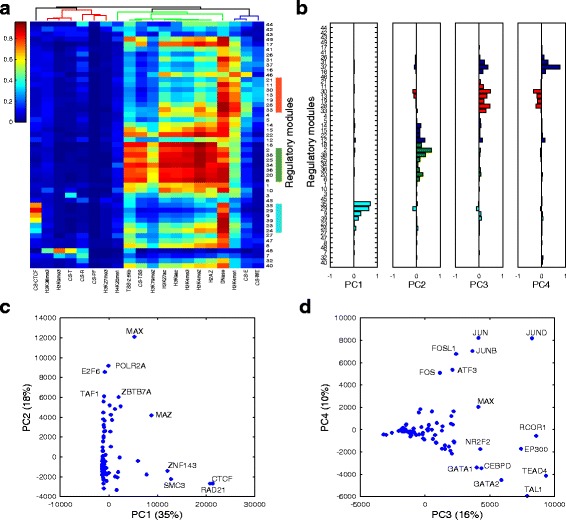
Epigenomic annotation and principal component analysis of the K562 regulatory modules. **a** A heatmap showing the fraction of regions using the same modules (*rows*) overlapping the specific annotations (*columns*). The modules (*rows*) are ordered as in Fig. 2. The top dendrogram was computed by applying hierarchical clustering on the fraction matrix with Pearson correlation distance and average linkage. **b** Principal component analysis (PCA) was performed on the factor-module matrix. The PCA loadings of the modules for the first four PCs. **c** The scatter plot shows the TF binding site counts projected on the principal components (PCs) 1 and 2. The percentage labels in the x and y axes represent the percentages of variance explained by the PCs 1 and 2, respectively. **d** Similar to **c**. The TF binding site counts projected on the PC3 and PC4. CS: chromatin state; T: transcribed region; R: repressed region; PF: promoter flanking region; TSS: promoter region including TSS; E: enhancer region; WE: weak enhancer or open chromatin region

We then examined whether the discovered regulatory modules were consistent with the chromatin states of the regulatory regions that use them. We annotated regulatory regions by DNase hyper-sensitivity, histone modifications, and the genome segmentation annotations derived from them [20, 42]. For the regulatory regions that utilize the same modules, we computed the fraction of the regulatory regions that overlap with these annotations. We found that for the regions using the same modules, the chromatin states of the regions are consistent with the functions of the TFs participating in the modules (Fig. 3a). For example, modules with master regulators of K562 cells and co-activator/co-repressors GATA1 + GATA2 + TAL1 + p300 + RCOR1 + TEAD4 are used in regions that are annotated with enhancer chromatin state and are enriched with H3K4me1 histone modification, while the Pol2/promoter modules are used in the regions that are annotated with TSS (transcription start site) chromatin state and are enriched with promoter-associated histone modifications such as H3K4me3, H3K9ac, and H3K27ac. Most regulatory modules are used by regulatory regions that are DNase hypersensitive, which may be explained by the preferential binding of TFs in open chromatin. Consistent with a previous study [21], the modules used in the non-DNase hypersensitive regions are pre-dominantly repressive modules with heterochromatin-bound factors, such as Module 48 (the combination of ZNF274, ZNF143, TRIM28, CBX3, SETDB1, and other factors) and Module 7 (the combination of ZNF143, TRIM28, CBX3 and SETDB1, but not ZNF274).

To further characterize the potential functions of the regulatory modules we discovered, we performed systematic gene ontology (GO) analysis using GREAT [31] on the genes proximal to the regions that use the same modules. We clustered the enriched GO terms based on the FDR q-values of the enrichment across the 49 modules. We found that specific GO terms are enriched in the regions using specific modules (Additional file 1: Figure S3). For example, housekeeping functions are enriched in promoter modules; regulatory functions and cell-type-specific terms such as “hematopoietic or lymphoid organ development” are enriched in both the enhancer and AP1 modules.

To facilitate interpretation, we further clustered modules that are driven by similar sets of TFs into 23 module groups. The modules in the same group share the same set of main TFs, yet differ in some specific minor TFs. For example, in the CTCF group, modules 29 and 35 both contain the TFs CTCF, RAD21, SMC3, and ZNF143; Module 29 includes ARID3A and CEBPB, while Module 35 includes MAX, ZBTB7A, MYC, and YY1.

RMD is able to discover widely used regulatory modules as well as very specific modules. For example, the promoter, enhancer, and CTCF modules are each used in more than 10,000 regulatory regions. At the same time, a small and specific module, ZNF274 + TRIM28 + SETDB1 (Module 48), which has been shown to specifically bind at the 3′ ends of zinc finger genes and suppress their expression [43], is used in only 63 regions.

To reveal the structure and importance of the discovered regulatory modules, we applied principal component analysis (PCA) to the module-TF matrix. We found that modules are reduced to principal components (PCs) that correspond well with the major module groups (Fig. 3b-d): the first PC is contributed mainly by the CTCF modules, explaining 35% of the total variance; the second PC is contributed mainly by the Pol2/promoter modules, explaining 18% of the total variance; the third and fourth PCs are contributed mainly by the p300/enhancer and AP-1 modules, explaining 16 and 10% of the total variance, respectively. In total, the first four PCs account for 79% of the total variance. These results indicate the dominant roles in genome-wide DNA binding of CTCF and cohesin, which have been suggested as key participants in shaping the three-dimensional genome structure [14], followed by promoter-binding factors and enhancer-binding factors. We found that the relative influence of the modules is not correlated with the number of factors in the modules because fewer factors contribute to the CTCF modules than to the promoter or enhancer modules.

### Regulatory module analysis identified distinct binding partners of NFY in different regions

In addition to identifying the global trends in the regulatory modules, the combinatorial patterns for certain groups of TFs also generate hypotheses about TF interactions. The modules we discovered reveal context-dependent co-binding as reported in previous work. For example, we found that FOS mainly participates in five modules (Additional file 1: Figure S4), which recapitulate four categories of FOS co-localization patterns reported previously [21]. Although the fifth FOS category “AP1-HOT” does not directly correspond to a single module, it can be factored into AP1 and promoter modules that are mixed in those regulatory regions. In addition, we found that the category of FOS + NFYB can be further divided into FOS + NFYB + SP2 (Module 40) and FOS + NFYB + USF (Module 32), which we discuss in more details below.

**Fig. 4:**
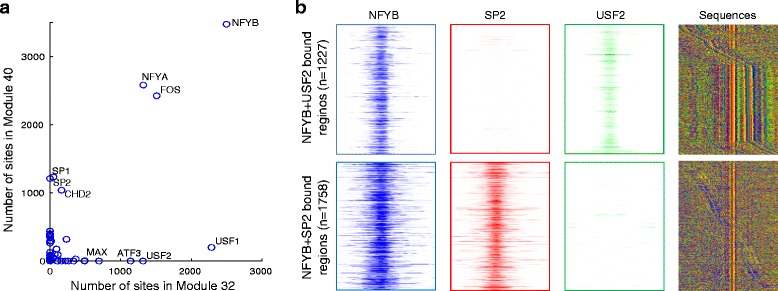
Regulatory module analysis identified distinct binding partners of NFY in different regions. **a** NFY (NFYA and NFYB) and FOS participate in two distinct modules. They co-bind with SP1 and SP2 in module 40 while in module 32, they co-bind with USF1 and USF2, ATF3 and other factors. The scatter plot shows the number of binding sites of each factor in the two modules. **b** NFYB binds exclusively with USF2 or SP2, each with different spacing constraints. The first 3 columns show ChIP-seq read enrichment of NFYB, SP2, and USF2 in a 1-kbp window around the NFYB binding sites. The 4th column shows sequence plots in a 100 bp window around the NFYB binding sites. The regions are centered at the NFYB binding sites and sorted by the distance between NFYB and USF2 or NFYB and SP2, respectively. The sequence motifs for USF2 and SP2 can be observed around the NFYB motifs. *Green*, *blue*, *yellow*, and *red* indicate base A, C, G, and T

NFYA and NFYB (two subunits of NFY) both participate in Modules 32 and 40, together with the co-binding partner FOS [21, 44]. However, in Module 32, NFY and FOS co-associate with USF1, USF2, ATF3, and MAX, while in Module 40, they co-associate with SP1 and SP2 (Fig. 4a). We verified that these two modules are used predominantly in different regions. More specifically, we found 1227 regions that are bound by both NFY and USF2 but not by SP2, 1758 regions that are bound by both NFY and SP2 but not by USF2, and only 120 regions that are bound by NFY, and both SP2 and USF2 (Fig. 4b). The majority of the regions using the NFY + SP module are TSS-proximal regions, while most of the NFY + USF regions are distal regions. Furthermore, NFY and USF2 co-binding exhibits a strong spacing constraint, with 1227 co-bound regions exhibiting a 21-22 bp spacing between NFY and USF2 motif-supported sites. On the other hand, NFY and SP2 co-binding does not appear to have a specific spacing constraint. Enrichment analysis using GREAT [31] shows that: the NFY + USF bound regions are near genes with ontologies such as “nuclear estrogen receptor alpha network pathway” (FDR q-value = 7.1E-5), “steroid hormone receptor binding” (FDR q-value = 2.1E-4) and “Homeobox protein, antennapedia type, protein family” (FDR q-value = 4.0E-4); while the NFY + SP bound regions are near genes with ontologies such as “Krüppel-associated box protein family” (FDR q-value = 2.3E-11), “positive regulation of nuclease activity” (FDR q-value = 1.6E-3) and “cholesterol biosynthesis” (FDR q-value = 3.5E-3). Taken together, the regulatory module analysis identified that NFY and FOS bind with distinct combination of factors in different types of regulatory regions that may regulate genes with distinct functions. NFY and SP1 have been shown to bind to promoters of a number of genes synergistically [45–47] or competitively [48]. NFY and USF co-bind as a complex to the promoter of HOXB4 gene in hematopoietic cells [49]. Here we find extensive NFY + SP and NFY + USF context-specific co-bindings occur in a mutually exclusive manner in thousands regions, suggesting the possibility of different DNA binding modes for NFY and different consequences of gene regulation. These results highlight that systematically discovered regulatory modules may be used to generate specific hypothesis that can be tested with more detailed analysis.

### The combinatorial rules of direct versus indirect binding

We next compared the co-binding partners of TFs when they bind directly (motif present) and indirectly (motif absent) to the genome. We define direct binding as the binding of a TF at sites that contain the cognate motif of the TF, and indirect binding as binding at sites that do not contain a detectable cognate motif. Previous studies have found that many ChIP-seq binding sites of sequence-specific TFs do not contain the cognate motif of the TFs, suggesting that binding may be indirect through the interaction with co-binding TFs [50, 51]. Understanding the combinatorial patterns of direct binding versus indirect binding may reveal the co-binding TFs that facilitate indirect binding.

**Fig. 5:**
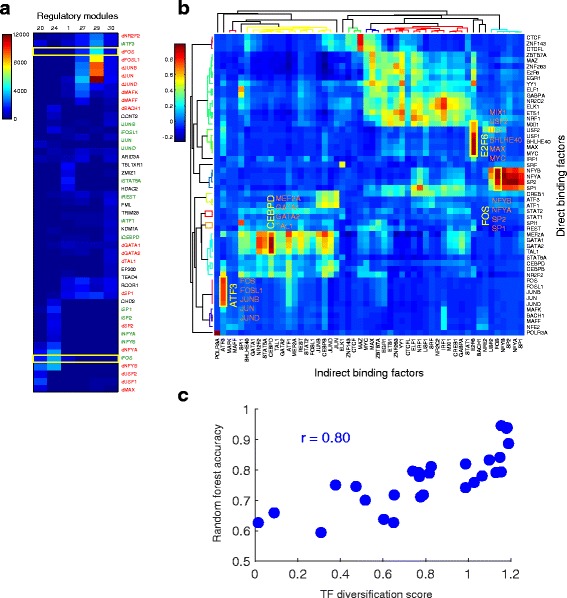
Direct versus indirect TF binding can be explained by the specific combination of co-binding TFs. **a** FOS co-binds with different set of TF partners when binding directly or indirectly. A subset of the regulatory module matrix that involves FOS binding are shown. Each cell in the heatmap represents the TF binding site count of a TF (*row*) in a module (*column*). dTF (*in red*) represents the direct binding sites. iTF (*in green*) represents the indirect binding sites. **b** RMD discovered specific combinations of TF indirect and direct binding. The heatmap shows the correlation between direct binding and indirect binding of 48 TFs based on their module participation. The top and left dendrograms were computed by applying hierarchical clustering on the correlation matrix with Pearson correlation distance and average linkage. *Yellow* boxes label combinations of TF indirect binding (*yellow text*) and direct binding (*orange text*). **c** Direct/indirect binding of some TFs can be predicted with high accuracy using the binding of co-binding TFs. A random forest classifier was trained to predict whether a TF binds directly or indirectly using the binding of the other TFs. Each point in the scatter plot is a TF. For a TF, a larger difference between the modules of direct binding and indirect binding corresponds to a higher the prediction accuracy by the random forest

For each sequence-specific factor X, the binding sites were divided into two groups: dX sites (direct binding) where X motif is present; iX sites (indirect binding) where X motif is not present. For example, CTCF sites were divided into dCTCF and iCTCF sites. These two groups were then treated as binding sites of distinct TFs. For the K562 dataset, this motif-based division expanded the total number of the TFs to 167, with 52 pairs of direct and indirect binding “factors” and 63 non-sequence-specific factors. Applying RMD to these data, we discovered 54 modules with the expanded set of direct and indirect factors (Additional file 1: Figure S5 and Additional file 2: Tables S5-S6). We then investigated the presence of co-binding factors that are specific to direct or indirect binding. For example, previous work reported that FOS co-localizes with NFYB [21]. Our analysis showed that this co-localization mostly occurs between indirect FOS binding and both direct and indirect NFYB binding (Modules 20 and 24) (Fig. 5a). To more systematically investigate the indirect binding of factors, we compute the pairwise correlation between all the 52 direct binding factors and all the 52 indirect binding factors across their module participation profiles (Fig. 5b). A high correlation means similar module participation, indicating that the indirect binding factor likely associates with the corresponding direct binding factor. We found several groups of such associations: indirect binding of FOS with direct binding of NFY + SP1 + SP2, indirect binding of E2F6 with direct binding of MYC + MAX + BHLHE40 + USF + MXI1 + YY1, and indirect binding of ATF3 with FOS + FOSL1 + JUNB + JUND + JUN.

Furthermore, we observed that the direct binding sites and indirect binding sites of some factors participate in very different modules. For example, direct FOS binding co-occurs with AP-1 factors (Module 29) and MAF + BACH1 + NFE2 (Module 27), while indirect FOS binding co-occurs with NFY + SP1 + SP2 (Module 24) or NFY + USF1 + USF2 (Module 20) (Fig. 5a).

With the observation that direct and indirect binding sites of some factors associate with different combination of factors, we reasoned that it would then be possible to predict whether a sequence-specific TF binds DNA directly or indirectly based on the proximal binding of other TFs. To test this hypothesis, we trained a random forest classifier to predict whether a TF binding site is a direct or indirect site using the proximal binding of other TFs in the region. We quantify the difference between the direct and indirect binding partners of a TF by introducing a TF diversification score, which is defined as the Pearson correlation distance between direct and indirect binding modules of the TF. For factors with high TF diversification scores, such as FOS, JUN, JUNB, JUND, MYC, SRF, USF1, and MXI1, the classifier predicted the direct/indirect binding of the factors with 80-95% accuracy (Fig. 5c and Additional file 4: Table S8). Furthermore, the ability to accurately predict direct and indirect binding of a TF is highly correlated to the TF diversification scores (Pearson correlation *r* = 0.80). The higher the TF diversification score, the higher the prediction accuracy of the random forest classifier (Fig. 5c). These results confirm that the direct and indirect binding of certain TFs can be explained by the specific combination of co-binding factors.

### Many regulatory regions use more than one module

**Fig. 6:**
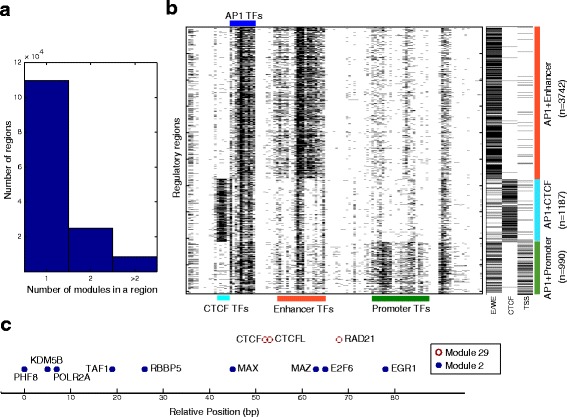
A large number of regulatory regions use multiple regulatory modules. **a** A histogram of the number of regulatory modules used in a regulatory region. **b** Co-occurring modules partition the regions that use AP-1 modules into distinct functional categories. *Left panel*: A heatmap showing the region-TF binding matrix of the regions co-bound by AP-1 and enhancer modules, AP-1 and CTCF modules, and AP-1 and promoter modules. The TFs are in the same order as in Fig. 2. *Right panel*: A heatmap showing the chromatin state annotation of the same regions. E/WE: enhancer/weak enhancer state; CTCF: CTCF state; TSS: TSS/promoter state. **c** An 80-bp region that contains binding sites assigned to a CTCF module (*color in brown*) and a promoter module (*color in blue*). Note that the positions of the sites assigned to different modules are mixed spatially

To understand the interplay among regulatory modules in regulatory regions, we investigated the extent of multiple-module usage by regions. The multiple-module usage is a structure that cannot be discovered by previous hard-clustering-based approaches, but can be revealed by RMD. We found that 25,107 regulatory regions (~18%) use more than one module (Fig. 6a). For example, we found 3,742 regions that use both enhancer and AP-1 modules (Fig. 6b), 3,071 regions that use both promoter and CTCF modules, and 2,514 regions that use both promoter and enhancer modules (Additional file 1: Figure S6). Notably, more than 63% the regions that use both enhancer and promoter modules are marked by both H3K4me1 (enhancer-related) and H3K4me3 (promoter-related) histone modifications (Additional file 1: Figure S6), but they are annotated as either TSS/promoter or enhancer/weak enhancer chromatin states [42]. This represents a limitation of genome annotation methods that only assign a single label to a genome segment. Furthermore, a module may co-occur with different other modules in distinct types of regulatory regions. For example, AP-1 modules co-occur with enhancer modules in the regions annotated with strong/weak enhancer chromatin states [42], co-occur with CTCF modules in regions mainly annotated with CTCF state, and co-occur with promoter modules in regions mainly annotated with TSS/promoter state (Fig. 6b). These different type of regions were also found to associate with different functional categories by a GREAT analysis [31]: the AP-1 and enhancer module co-bound regions are enriched with genes involved in “platelet activation” (FDR q-value = 3.1E-11), “regulation of inflammatory response” (FDR q-value = 4.4E-11), “regulation of translation” (FDR q-value = 6.3E-10), and “myeloid leukocyte activation” (FDR q-value = 1.1E-7); while the AP-1 and promoter module co-bound regions are enriched with genes involved in “viral process” (FDR q-value = 1.4E-12), “protein kinase binding” (FDR q-value = 3.7E-11), and “apoptotic signaling pathway” (FDR q-value = 3.3E-9). These differences in functional enrichment suggest that AP-1 module carries out distinct functions when it is combined with different type of other modules.

To ensure that the discovery of multiple-module usage was not the result of inappropriate merging of proximal regulatory regions, we chose a more conservative inter-site distance, 50 bp, for merging the sites into regions. Furthermore, we studied the positions of the binding sites that are assigned to different regulatory modules. In many cases, the binding sites assigned to distinct modules are spatially mixed. For example, in an 80 bp region on chromosome 1, CTCF, CTCFL and RAD21 sites from a CTCF module are mixed with POL2, E2F6, MAX, EGR1, and other sites from a promoter module (Fig. 6c). Such co-occurrences between CTCF and promoter modules are consistent with previous findings that CTCF mediate long-range DNA-looping interactions between enhancers and promoter [13], and that ZNF143 binds directly to the promoters and occupies anchors of chromatin interactions connecting promoters with distal enhancers [36].

In summary, our analysis suggests that multiple-module usage is a prevalent aspect of regulatory activities in the cells and it is revealed by RMD.

### Cell-type-specific regulatory modules

**Fig. 7:**
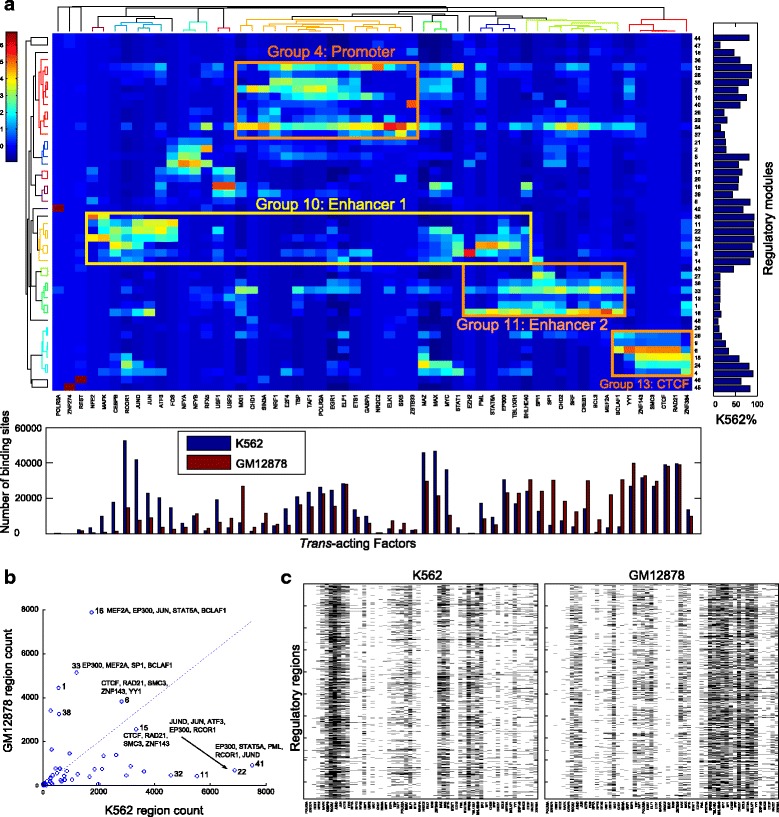
Common and cell-type-specific regulatory modules. **a** RMD was applied to ChIP-seq binding sites of 56 TFs in human K562 cells and GM12878 cells. Each cell in the heatmap represents the z-score of TF binding site count (standardized along the columns) of a TF (*column*) in a module (*row*). The bottom bar plot shows the total number of binding sites of the TFs. The right bar shows the percentage of the sites contributed from K562 data for the modules. The top and left dendrograms were computed by applying hierarchical clustering on the regulatory module matrix with Pearson correlation distance and average linkage. **b** A scatter plot showing that the cell-type-specific modules are preferentially used in the differentially bound regions in K562 cells and GM12878 cells. **c** Distinct cell-type-specific modules are used in the same genomic regions in different cell types. A set of 1956 regions that are bound by K562 enhancer module factors in K562 (*left panel*) and by GM12878 enhancer module factors in GM12878 cells (*right panel*). The TFs are in the same order as in **a**

We next investigated if we could observe cell-type-specific and cell-type-common regulatory modules. We used ChIP-seq data of 56 TFs that were profiled in both K562 and GM12878 cells [20]. Following a previous approach [21], we constructed the co-binding regions in each cell type separately (~105,000 regions in K562 and ~91,000 regions in GM12878) and then combined the data from all regions from both cell types for RMD analysis. RMD discovered 48 modules that describe the binding of the 56 factors in K562 and GM12878 cells (Fig. 7a). To aid interpretation of the modules, we clustered them into 16 module groups. Interestingly, the promoter-associated modules and CTCF-associated modules are each clustered into one group that is common to K562 and GM12878, while the enhancer-associated modules are clustered into two cell-type-specific groups.

To investigate the degree of cell-type-specificity of the discovered modules, we computed the fraction of the binding regions that are contributed from the K562 data or from the GM12878 data for each module. We found that some combinations of factors are mainly used in K562 cells and others are mainly used in GM12878 cells even though all of the 56 factors bind regulatory regions in both cell types (Fig. 7a). In particular, many enhancer modules are preferentially used in one cell type. In K562 cells p300 co-binds with JUND, JUN, RCOR1, ATF3, FOS, CEBPB, MAFK, MAX, and MYC (Module group 10), but in GM12878 cells p300 co-binds with MEF2A, SP1, SPI1, BCLAF1, BCL3, and BHLHE40 (Module group 11). Both cell types share other module groups, such as the promoter and CTCF module groups. In the regulatory regions that are bound in K562 or GM12878 cells but not bound in both cell types, cell-type-specific modules are used preferentially by one cell type, while shared modules are used in both cell types (Fig. 7b).

We next investigated if a regulatory region that is bound in both cell types uses cell-type-specific regulatory modules. Out of 50,910 regions that are bound in both cell types, we found that 1,956 regions use a different enhancer module in the two cell types (Fig. 7c). For these 1,959 regions, module group 10 is used in the K562 cells and module group 11 is used in the GM12878 cells. Thus although these regions are bound in both cell types and may act as enhancers, as suggested by the binding of transcriptional co-activator p300, they are bound by cell-type-specific combinations of factors in K562 and GM12878. In addition, we found 1,312 regions that are bound by CTCF module factors in both K562 and GM12878 cells, and also bound by the K562 enhancer module factors in K562 cells, suggesting the usage of K562-specific enhancers in these CTCF/cohesin bound regions. In summary, important differences can exist between the set of regulatory modules that bind the same regulatory regions in distinct cell types.

## Conclusions

Gene regulation specificity is orchestrated by the interactions among a complex group of *trans*-acting factors that we have organized into distinct combinable modules. Previous methods have modeled TF combinatorial binding as pairwise interactions or as a single module at a given regulatory region; they are thus not able to capture the complexity of modular combinatorial binding. We have developed a new approach to summarize high-dimensional binding data into combination of combinatorial binding modules. Our approach can provide important insights into the mechanisms of gene regulation not available with previous hard-clustering-based methods. The modules discovered are easy to interpret individually and as a whole, capturing key aspects of global combinatorial binding patterns and providing a resource for generating new hypotheses for TF interactions.

Our analysis reveals that modular combinatorial binding occur in tens of thousands of regions and that specific combination of modules may regulate distinct functional groups of genes, suggesting that multiple-module usage is a prevalent aspect of regulatory activities in the cells. Modeling TF combinatorial binding as regulatory modules helps to dissect the complexity of combinatorial binding of many TFs into compact and easily interpretable modules. Moreover, such explicit modeling of modular structure helps to uncover specific modules that are combined with other modules and are easy to be missed by previous approaches. With a larger number of additional TFs being assayed by large-scale efforts such as the ENCODE project [20], we expect that RMD will be useful in revealing the complexity of combinatorial binding in these future data.

Previous work attempted to distinguish between direct and indirect TF in vivo binding by integrating in vivo nucleosome occupancy data and in vitro protein binding microarray experiments [51], or by using TF binding motifs and DNase-seq footprints [52]. In this work, we discovered that the direct and indirect DNA binding of certain TFs is associated with distinct sets of co-binding partners and that without using motif information the co-binding partners alone can predict whether the TF binds directly or indirectly with high accuracy. In addition, our direct/indirect combinatorial binding maps allow prediction of co-binding TFs that may facilitate the indirect binding of TFs. The direct/indirect binding analysis was conducted with a simplified classification of the binding sites based on whether they contain a detectable cognate motif. Recent studies show that clusters of low-affinity binding sites with degenerate motifs can be functional [53] and that binding sites without consensus motifs may use DNA shape to facilitate the in vivo binding of TFs [54]. Future analyses that take into account the clustering of binding sites and the DNA shape information may gain more insights on the role of combinatorial binding of co-binding factors on TF binding.

Our method is a general method for studying TF combinatorial binding. It can be applied to various cell types and species [11, 12, 55, 56] where sufficient binding data are available. One potential limitation on studying combinatorial TF binding from ChIP-seq data is the relative scarcity of high quality antibodies. To expand RMD combinatorial binding analysis to more TFs or to cell types that do not have sufficient ChIP-seq data, one strategy is to augment or replace ChIP-seq data with TF binding predicted from DNase-seq [57] or ATAC-seq [58] data and TF motif information [59, 60].

## Methods

### Data and preprocessing

ChIP-seq data for the TFs and corresponding controls were downloaded from the ENCODE project website http://hgdownload.cse.ucsc.edu/goldenPath/hg19/encodeDCC/. Fastq files were aligned to hg19 genome with Bowtie [61] version 0.12.7 with options “-q --best --strata -m 1 -p 4 --chunkmbs 1024”. GEM [62] was used to call binding events with default parameters using the aligned reads of TF ChIP-seq experiments and the corresponding control experiments. GEM produces two set of binding site calls for each dataset: GPS binding calls without motif information and GEM binding calls with motif information. The binding calls overlapping with the ENCODE blacklist regions (http://hgdownload.cse.ucsc.edu/goldenPath/hg19/encodeDCC/wgEncodeMapability/wgEncodeDacMapabilityConsensusExcludable.bed.gz) were excluded for this analysis.

### Construct co-binding regions

GPS binding calls of all the factors in a given cell type were pooled together to construct the co-binding regions. Each binding call was expanded +/−50 bp from the summit position. Then overlapping binding calls were merged to form non-overlapping co-binding regions. Only co-binding regions with three or more binding calls were used for subsequent analysis. We also performed analyses using regions that have a minimum of 2 or 4 TF sites, the results are similar (data not shown). An alternative binding site expansion distance of 100 bp was tested to construct co-binding regions; it gives similar results. For this paper, the expansion distance of 50 bp was used because the spatial resolution of TF ChIP-seq binding calls is about 30-50 bp and that 50 bp expansion distance is more conservative for analyzing multi-module co-occurrences than the 100 bp distance. From the K562 co-binding regions (*n* = 142,962), we construct a Region-TF matrix (142,962 × 115) that contains the number of binding sites of each TF in each region. The code for constructing co-binding regions and for generating topic model input files is freely available at http://groups.csail.mit.edu/cgs/gem/rmd/.

### Topic model

The hierarchical Dirichlet processes (HDP) topic model was used in this study because it automatically determines the number of the topics from the data. A C++ implementation of HDP was downloaded from http://www.cs.columbia.edu/~blei/topicmodeling_software.html. The parameters used were “--eta 0.1 --max_iter 2000”. Eta is the hyperparameter for the topic Dirichlet distribution. We tested different eta values (0.01, 0.05, 0.1, 0.5 and 1) and the results were similar. We chose eta to be 0.1 to encode our assumption that each topic contains only a few TFs. The HDP inference procedure typically converged at about 1000 iteration. We ran the HDP with 3 different random seeds for 2000 iterations and used the run that had the highest data likelihood reported by the HDP. The input to the HDP are the TF binding site counts in the co-binding regions. Each region is treated as a document and the TF sites as words in the documents. The output of the HDP includes the module-TF matrix and the module assignment of each TF binding site.

For the module-TF matrix, each column vector (TF participation vector) describes the distribution of the TF binding sites across all the modules, and each row vector (module vector) represents the number of binding sites contributed by each TF to the module. We compute a z-score for each TF vector. A TF is considered to participate a module if the z-score of the TF-module pair is larger than 1. Similarly, we compute a z-score for each module vector. A TF is considered to be a “main driver” of the module if the z-score of the TF-module pair is larger than 1. Each module is labeled with the names of the main TF drivers, which are ranked by their z-scores. To facilitate interpretation of the modules, the module-TF matrix was clustered into module groups using hierarchical clustering with Pearson correlation distance and average linkage. The cutoff distance for clustering is 0.5.

The region-module assignment table assigns each TF binding site from each region to one of the modules. The assignment table was summarized into a region-module matrix where each element of the matrix represents the number of the TF binding sites in a region that are assigned to a particular module. A module is considered as being used in a particular regulatory region if 1) at least three binding sites in the region are assigned to the module and 2) the z-score of the site count for the module in the region is larger than 1.

### Comparing HDP with k-means clustering and NMF

To test the ability of RMD, k-means clustering, and NMF to accurately capture TF-TF correlations that are present in the binding data, we applied all three approaches to the same set of K562 cell ChIP-seq binding data to discover the same number of modules (k = 49). We applied k-means clustering and NMF on the K562 Region-TF binding matrix using the MATLAB software (MATLAB and Statistics Toolbox Release 2012a, The MathWorks, Inc., Natick, Massachusetts, United States). For k-means clustering, Euclidean distance is used as the distance metric. The cluster number (i.e. rank for NMF) was set to be k = 49 and k = 100 to compare with the HDP topic model with 49 topics. We refer the k-means clusters, NMF components and HDP topics as the modules. To compare the three approaches, we first computed the pairwise TF correlation scores using the original region-TF matrix, or the module-TF matrices derived from these three methods. Then we computed the correlation between the pairwise TF correlation scores from the original region-TF matrix and those from the three derived module-TF matrices. We also compared topic model modules and k-means clusters by computing their Pearson correlation using the module vectors versus the cluster vectors. The comparison results are robust across different correlation cutoff values for matching the modules (Additional file 1: Figure S1c).

### Principal component analysis (PCA)

PCA was performed using the MATLAB software (MATLAB and Statistics Toolbox Release 2012a, The MathWorks, Inc., Natick, Massachusetts, United States) on the module dimension of module-TF matrix.

### Protein-protein interaction and epigenomic annotation of regulatory modules

The protein-protein interaction derived from IP-MS Data for K562 cells [21] was downloaded from http://www.cell.com/cms/attachment/2021777707/2041662737/mmc1.xls. For the 33 direct physical interaction pairs that contain the TFs in our study, we considered an interaction as rediscovered by RMD if the two TFs are both the “main drivers” in a same module. The p-value of overlap between IP-MS data and the RMD modules was calculated by fixing the pulled-down TFs while permuting all the partners identified by mass spectrometry and calculating the odds of getting higher overlap with RMD modules. A total of 200 permutations were performed, enabling us to estimate the p value to the level of 0.05.

DNase hyper-sensitive open chromatin peak calls, histone modification peak calls, and the combined genome segmentation annotations were downloaded from http://ftp.ebi.ac.uk/pub/databases/ensembl/encode/integration_data_jan2011/. The co-binding regions were annotated with the epigenomic annotations if they overlap at least 1 bp. For each module, we identified the regulatory regions that use the module and computed the fractions of these regions that overlap with the annotations.

### GREAT ontology enrichment analysis

The GREAT ontology enrichment analysis [31] was performed on the GREAT website (http://bejerano.stanford.edu/great/public/html/index.php) with the default “basal plus extension” association rule. BED files of the regions that use the regulatory modules are used as the inputs. Hierarchical clustering was performed to cluster the GO terms on the -log_10_ (FDR q-value) of GO terms with Pearson correlation distance and average linkage.

### Direct versus indirect binding analysis

For the direct versus indirect binding analysis, GEM binding calls were used for sequence-specific binding factors that the GEM motifs can be verified. The positional frequency matrix of the top ranked motif reported by GEM was compared against known motifs of the same factor in the public databases using STAMP [63], as previously described [62]. For the 52 sequence-specific TFs that a database match for the top motif is found, the GEM binding calls were divided into direct and indirect binding sites based on whether the binding sites contain a motif match of the TF. The direct and indirect binding sites were treated as separate factors for topic modeling analysis. For example, CTCF sites were divided into dCTCF and iCTCF sites. For the non-sequence-specific factors and sequence-specific factors that the top GEM motif does not match the known database motifs of the factor, GPS binding calls were used. All the GEM and GPS binding calls were then pooled together to construct the co-binding regions. In total, 159,204 co-binding regions with binding sites from 167 “factors” were constructed. Applying RMD, we discovered 54 modules. The correlation between direct binding of a TF and indirect binding of another TF (matrix shown in Fig. 5b) were computed using the TF vectors in the module-TF matrix.

### Predicting direct/indirect binding using random forest

We trained a random forest (RF) classifier to predict whether a TF binding site is a direct or indirect site using the proximal binding of other TFs in the co-binding region. We used the TreeBagger implementation of RF in the MATLAB software (MATLAB and Bioinformatics Toolbox Release 2015b, The MathWorks, Inc., Natick, Massachusetts, United States). More specifically, using the region-TF matrix (159,204 × 167), we took the rows that contained either direct or indirect binding sites of the TF, used the columns corresponding to the direct or indirect binding of the TF as the prediction target and the rest of the columns (binding of the other TFs) as the features. For each sequence specific co-binding TF, the dTF and iTF columns were combined, ignoring the motif information. Therefore, the prediction is harder because it based only on the identity but not the motif information of the co-binding TFs. For each sequence-specific TF, we trained five RFs, each with a distinct random subset (80%) of the data, and then tested on the rest of 20% data. The prediction accuracy values of the five classifiers are then averaged for each TF. The correlation between direct and indirect binding of a TF (shown in Fig. 5c) were computed using the corresponding TF vectors in the module-TF matrix.

### Cross-cell-type analysis

We used ChIP-seq data of 56 TFs that were profiled in both K562 and GM12878 cells [20]. When there were multiple datasets for the same factor, we chose the datasets that were produced by the same lab, using the same antibodies, or had similar number of binding calls. Following a previous approach [21], the co-binding regions were constructed separately for K562 and GM12878. These data from both cell types were then concatenated for topic model analysis. Thus we can learn TF co-binding relationships that are shared across cell types but still keep track of the cell type origin of the TF sites and the regions.

## Additional files

Additional file 1: Figures S1-S6. (PDF 1964 kb)
Additional file 2: Tables S1-S6. The RMD module matrix and summary for K562, GM12878, and K562 direct versus indirect binding, respectively. (XLS 181 kb)
Additional file 3: Table S7. The overlap between RMD modules and published protein-protein interactions. (XLS 25 kb)
Additional file 4: Table S8. TF diversification scores and random forest prediction accuracies. (CSV 609 bytes)

## Abbreviations

HDP: Hierarchical dirichlet processes
IP-MS: Antibody immunoprecipitation and mass spectrometry
NMF: Non-negative matrix factorization
PCA: Principal component analysis
RF: Random forest
RMD: Regulatory module discovery
SOM: Self-organizing maps
TF: *Trans*-acting factors
TSS: Transcription start site
