# Supplementary material for: Modular combinatorial binding among human trans-acting factors reveals direct and indirect factor binding
Source: BMC Genomics. 2017 Jan 6;18:45. doi: 10.1186/s12864-016-3434-3 (PMC5219757; doi:10.1186/s12864-016-3434-3)
Supplement: Additional file 1: Figures S1-S6. — (PDF 1964 kb) [file 12864_2016_3434_MOESM1_ESM.pdf]

Supplemental Figures for:

Modular Combinatorial Binding among Human *Trans*-acting Factors Reveals Direct and Indirect Factor Binding

Yuchun Guo[1], David K Gifford[1]

[1]MIT, Computer Science and Artificial Intelligence Laboratory, Cambridge, MA 02139, USA

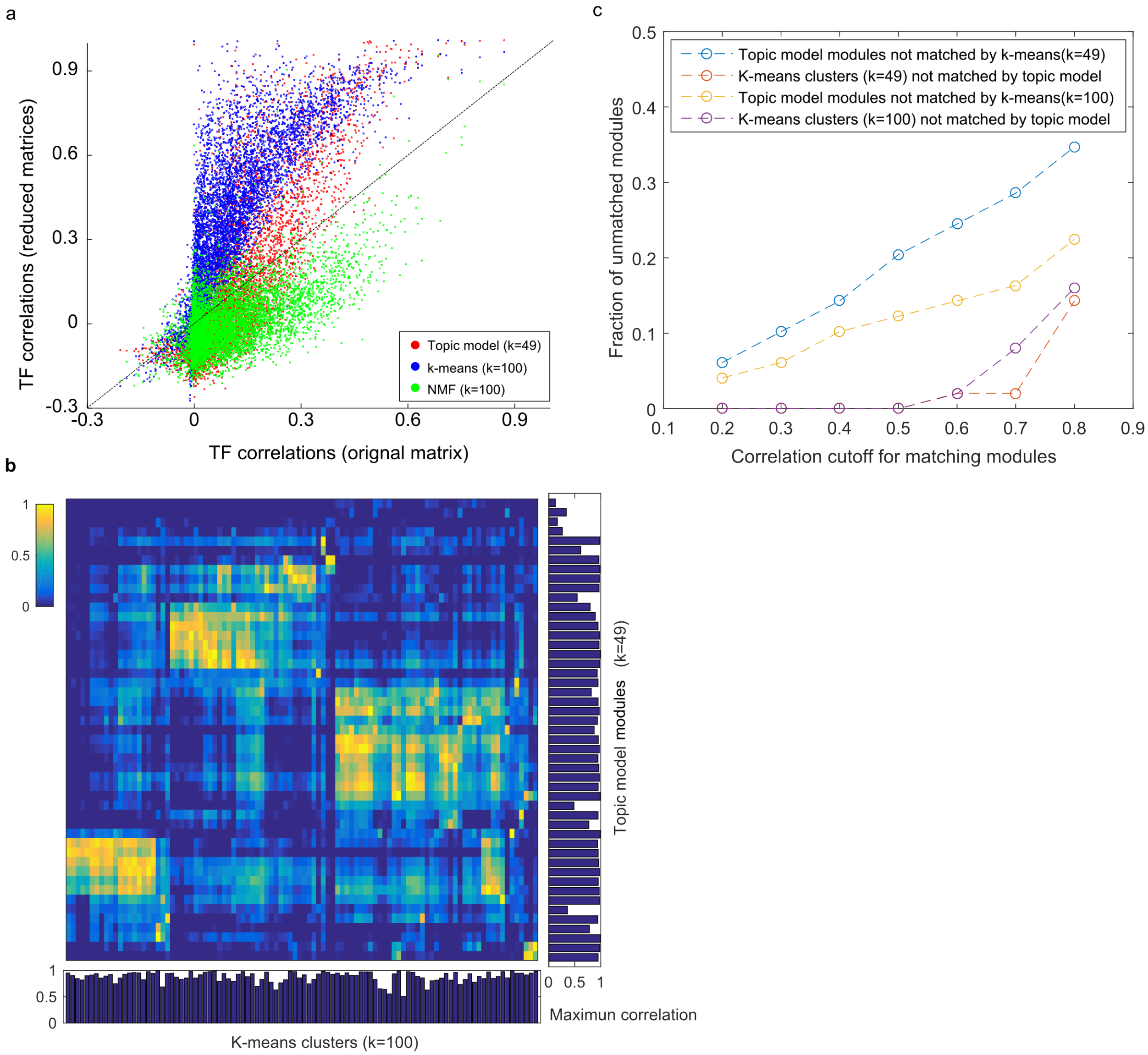

Figure S1. RMD is able to discover modules that are not discovered by k-means clustering.

a The topic model re-capitulates the original binding data more accurately than k-means clustering and NMF. Each point in the scatter plot represents the Pearson correlation coefficient between a pair of TFs calculated using the original binding data (x-axis) or calculated using the reduced data matrix (y-axis) by topic model (k=49), k-means clustering (k=100), or NMF (k=100). **b** A heatmap shows the Pearson correlations between topic model regulatory modules (k=49) and k-means clusters (k=100). The modules are ordered as in Fig. 2. Bottom bar chart shows the maximum correlation values for each k-means cluster. All k-means clusters are matched by at least one modules, with a maximum correlation value larger than 0.5. Right bar chart shows the maximum correlation values for each module. Six modules cannot be matched by any k-means clusters. **c** The fractions of unmatched modules at various cutoffs for correlation values. A larger fraction of topic model modules are not matched by the k-means clusters (k=49 or k-100) than the fraction of the unmatched k-means clusters.

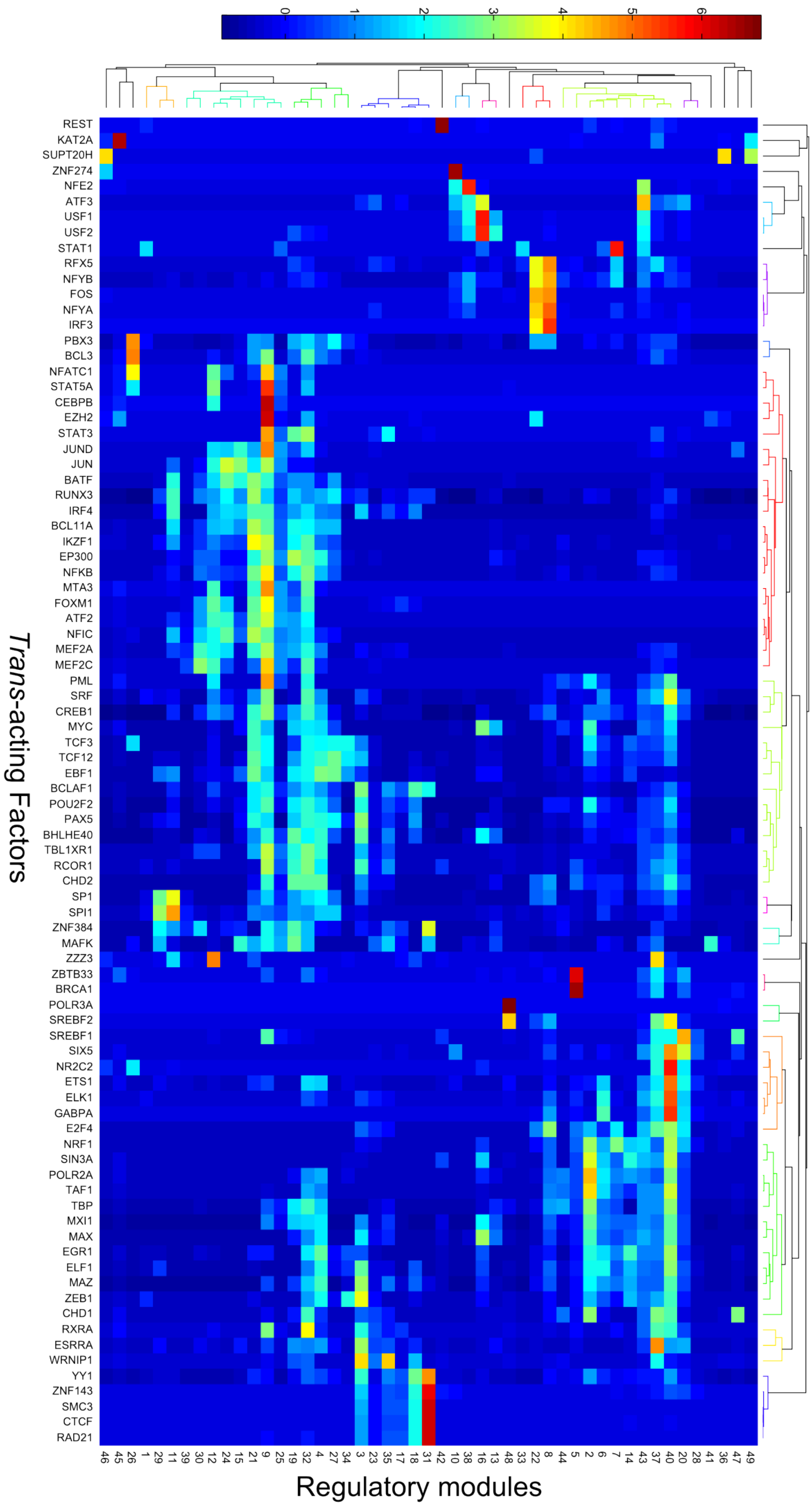
0
1
2
3
4
5
6
Trans-acting Factors
REST
KAT2A
SUPT20H
ZNF274
NFE2
ATF3
USF1
USF2
STAT1
RFX5
NFYB
FOS
NFYA
IRF3
PBX3
BCL3
NFATC1
STAT5A
CEBPB
EZH2
STAT3
JUND
JUN
BATF
RUNX3
IRF4
BCL11A
IKZF1
EP300
NFKB
MTA3
FOXM1
ATF2
NFIC
MEF2A
MEF2C
PML
SRF
CREB1
MYC
TCF3
TCF12
EBF1
BCLAF1
POU2F2
PAX5
BHLHE40
TBL1XR1
RCOR1
CHD2
SP1
SPI1
ZNF384
MAFK
ZZZ3
ZBTB33
BRCA1
POLR3A
SREBF2
SREBF1
SIX5
NR2C2
ETS1
ELK1
GABPA
E2F4
NRF1
SIN3A
POLR2A
TAF1
TBP
MXI1
MAX
EGR1
ELF1
MAZ
ZEB1
CHD1
RXRA
ESRRA
WRNIP1
YY1
ZNF143
SMC3
CTCF
RAD21
46 45 26 1 29 11 39 30 12 24 15 21 9 25 19 32 4 27 34 3 23 35 17 18 31 42 10 38 16 13 48 33 22 8 44 5 2 6 7 14 43 37 40 20 28 41 36 47 49
Regulatory modules

Figure S2. RMD discovered 49 regulatory modules using GM12878 TF binding data

RMD was applied to a compendium of ChIP-seq binding sites of 86 TFs in human GM12878 cells and discovered 49 regulatory modules. Each cell in the heatmap represents the z-score of the TF binding site count (standardized along the columns) of a TF (column) in a regulatory module (row). The top and left dendrograms were computed by applying hierarchical clustering on the regulatory module matrix with Pearson correlation distance and average linkage.

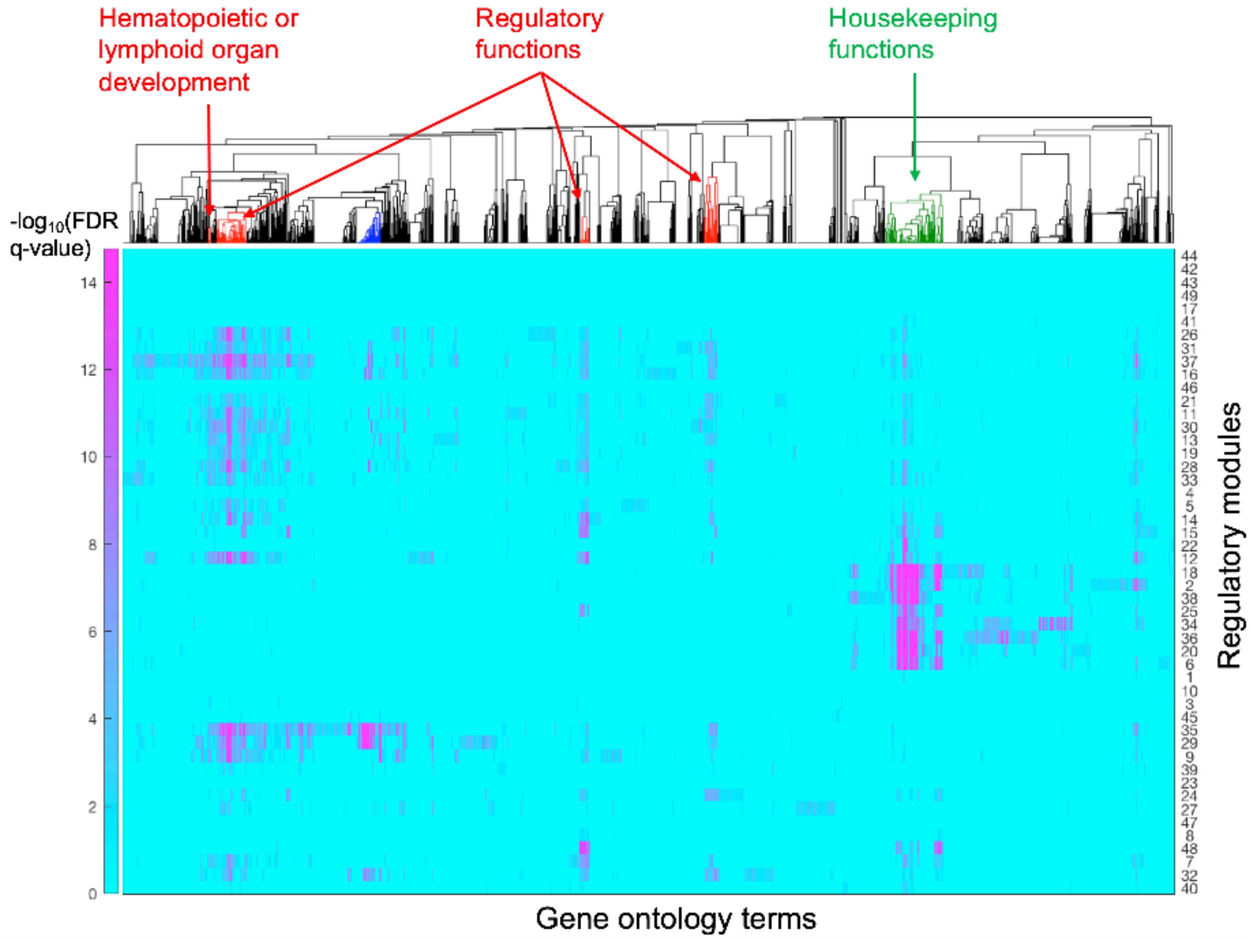

Figure S3. The GO term enrichment in regulatory modules.

The enriched GO terms are obtained from GREAT analysis on the regions that use the regulatory modules. The top dendrogram was computed by applying hierarchical clustering on the -$\log_{10}$(FDR q-value) of GO terms with Pearson correlation distance and average linkage. The regulatory modules (rows) are ordered as in Figure 2.

a

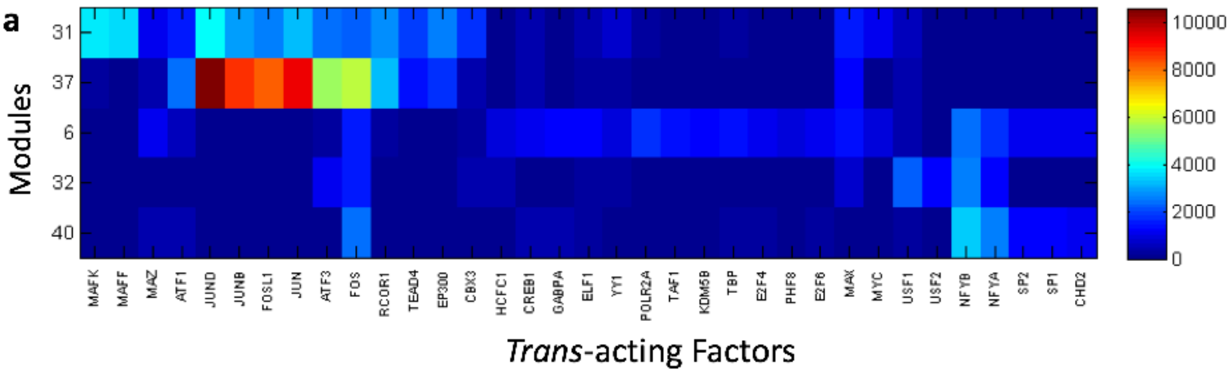

b

Modules	Xie et. al categories
31	EP300 mediated distal
37	Canonical AP1
6	Proximal - HOT
32	FOS+NFYB
40	FOS+NFYB
37 + 6	AP1 - HOT

Figure S4. The regulatory modules reveal context-dependent FOS co-binding as reported in previous work.

a A subset of the regulatory module matrix that involves FOS binding are shown. Each cell in the heatmap represents the TF binding site count of a TF (column) in a regulatory module (row). **b** The modules recapitulate five categories of FOS co-localization patterns reported previously (Xie et al., 2013).

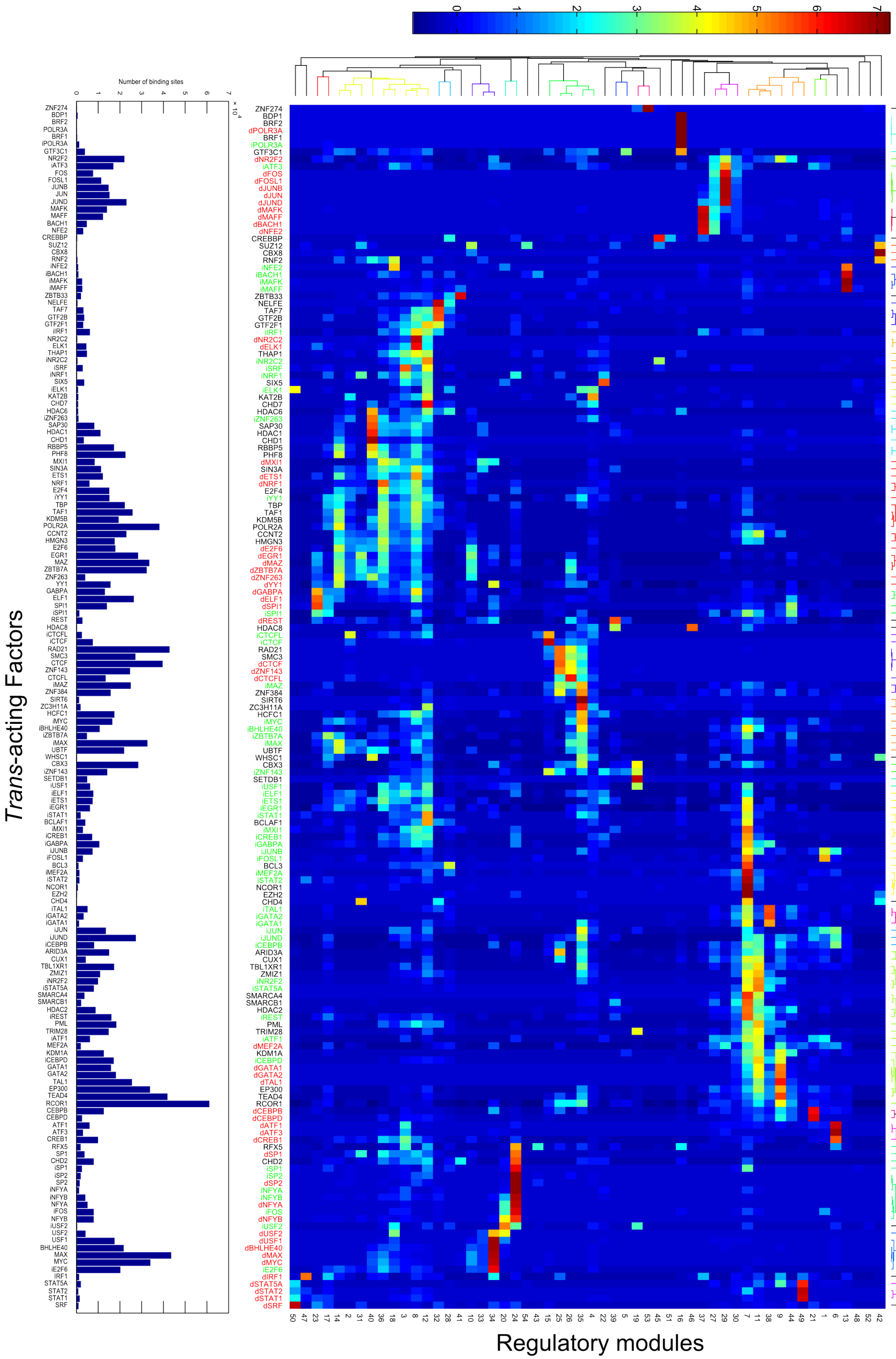

Number of binding sites
Trans-acting Factors
Regulatory modules

Figure S5. The direct/indirect binding regulatory modules in K562 cells

RMD was applied to a compendium of ChIP-seq binding sites of 167 direct and indirect binding "factors" in human K562 cells and discovered 54 regulatory modules. Each cell in the heatmap represents the z-score of the TF binding site count (standardized along the columns) of a TF (column) in a regulatory module (row). The factor names are colored according to their binding types: direct binding factors dTF (red), indirect binding factors iTF (green) and factors with unknown binding type (black). The top and left dendrograms were computed by applying hierarchical clustering on the regulatory module matrix with Pearson correlation distance and average linkage. The bottom bar plot shows the total number of binding sites of the "factors".

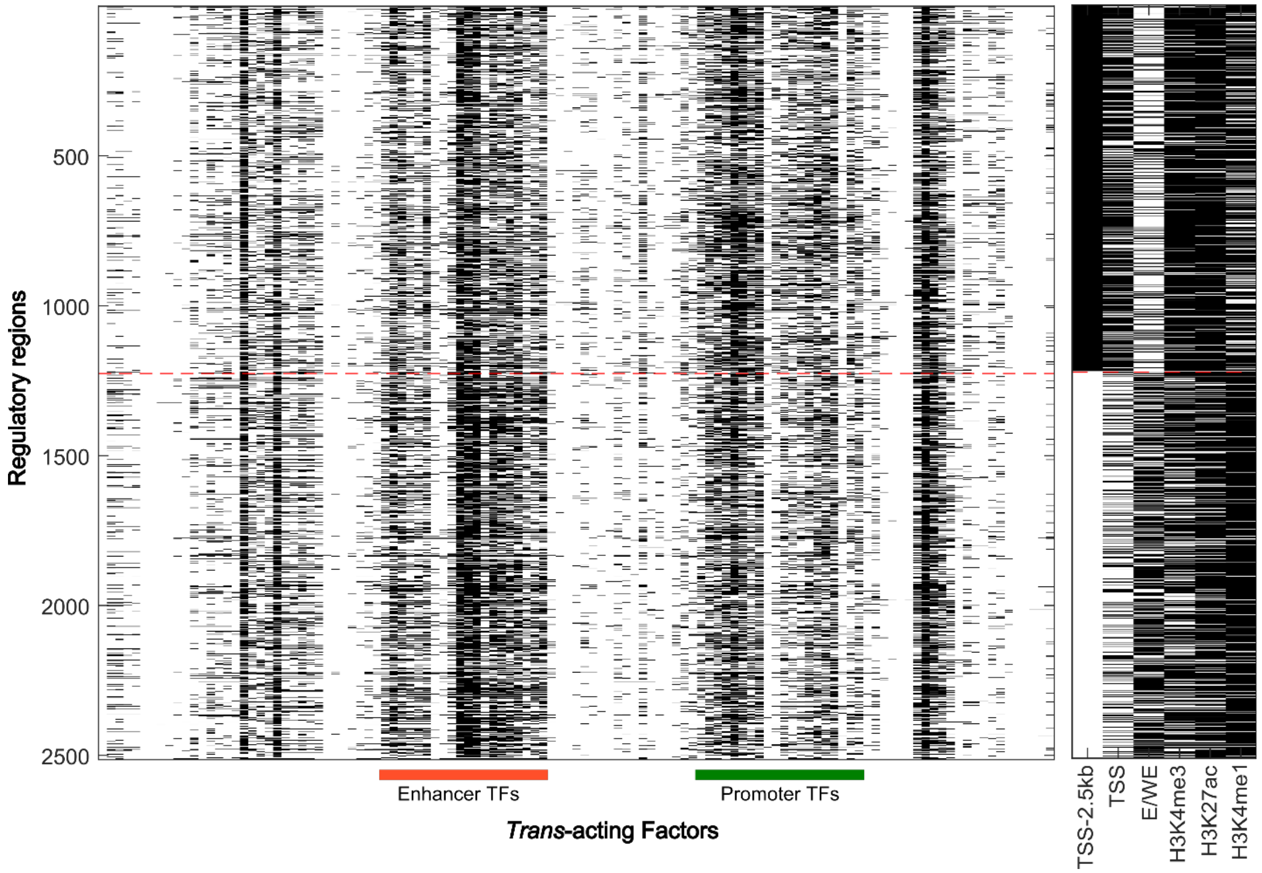

Figure S6. Co-occurrence of enhancer and promoter modules.

Left panel: A heatmap showing the region-TF binding matrix of the 2514 regions co-bound by enhancer and promoter modules. There are 30477 regions bound by enhancer modules and 27349 regions bound by promoter modules (not shown). The regions are divided into those that are within 2.5kb of the TSSs and those that are not within 2.5kb of the TSSs. The TFs are in the same order as in Figure 2. Right panel: A heatmap showing the chromatin state and histone mark annotations of the same regions. E/WE: enhancer/weak enhancer chromatin state; TSS: TSS/promoter chromatin state.
